# Pitfalls in scientific research: critical appraisal of articles published in one of the international journals in Egypt

**DOI:** 10.1186/s43046-020-00050-4

**Published:** 2020-10-26

**Authors:** Sarah S. Nasr, Ghada M. Sherif, Amal S. Ibrahim, Rasha M. Allam

**Affiliations:** National Cancer Institute, Cairo University, Cairo, Egypt

**Keywords:** Critical appraisal, Peer-review, Scientific articles, Medical manuscript review, Study design, Statistical methodology

## Abstract

**Background:**

To identify and report flaws of Internet-published articles in the *Journal of the Egyptian National Cancer Institute* (JENCI), Cairo University, through a retrospective documentary study on articles published during the period from 2011 to 2016. All sections were reviewed against a collective checklist. Articles were grouped by publication year into 2 intervals: early (from 2011 to 2013) and recent (from 2014 to 2016) to identify changes in study characteristics over time.

**Results:**

The study included 139 original articles. Half of the titles represented aim and 9.4% represented study design. Abstracts were concise, clear, with structured writing format in 98.6%, 93.5%, and 35.3%, respectively. Most introductions included the study aim, while 41% had a rationale. Study timing was reported in 59.0%, while the study design was reported in 25.9%. Inclusion and exclusion criteria were clearly reported in 43.1% and 40.1%, respectively. Statistical methods were mentioned in 80.6%, complete in 30.4%, and appropriate in 85.7%. Four studies reported sample size estimation. Only 52.5% and 58.3% of results were exhaustive and answer the research question, respectively. Incorrect statistical calculations occurred in 41.0%, inappropriate statistical tests or descriptive parameter selection in 26.6%, while inappropriate test application occurred in 49.1%. About 60% of discussions did not completely cover results, 31.7% fully justified the findings, 56.1% followed a logical flow, and 36.7% had contradiction within the text. Conclusions were mostly linked to aim, imprecise, and extrapolating beyond results. On comparing both periods, only a significant less misuse of statistical terms, more reporting conflict of interest, more missing references for cited texts in the recent period, and more participation of NCI over other institutes in the early period were found.

**Conclusion:**

Articles published in JENCI (from 2011 to 2016) had many methodological and reporting defects and some points of strength. Using the collective checklist developed by this study, continuous training of researchers, involving epidemiologists throughout the whole research process, and applying strict journal reporting and publication rules should be encouraged.

## Background

In the medical field, research papers provide information on current practice and new developments in the diagnosis, prevention, and treatment of disease. It is crucial to the development and application of evidence-based healthcare through the integration of best evident practice, clinical experience, and patient preference [1].

Doing scientific research is half the work done, but no matter how spectacular its results are, it is not completed until these results are published. Unfortunately, the education of scientists is mainly directed towards the technical aspects of science, while the communication arts are neglected or ignored or in other words, many good scientists are poor writers. [2]

Critical appraisal is the use of explicit methods to assess the data in published research, applying the rules of evidence to factors such as internal validity, adherence to reporting standards, conclusions, and generalizability [3].

Defects in scientific manuscripts include the basic structure of the study starting from the appropriateness of the study design up to the proper use of statistical analysis. This highlights the urgent need for the involvement of a well-trained epidemiologist from the start. [4]

The *Journal of the Egyptian National Cancer Institute* started publishing articles in 1985 with 2 issues per year, continued after 1997 with 4 issues per year, then became available online through PubMed (2005), Elsevier (2011), and SpringerOpen (2019) databases.

Literature lacks references to prior efforts of articles review after-publication, so this study was necessary to ensure continuous improvement in research quality and help authors learn from common pitfalls. The goal of this study was to identify the more frequently committed flaws of the Internet-published scientific papers in JENCI (in the period from 2011 to 2016) to shed light on areas that might need improvement and to evaluate how the quality of medical research has changed over time.

## Methods

This is a retrospective documentary study. A peer-reviewed data collection form was constructed using a set of published scientific reporting guidelines [4-13].

### Eligibility criteria

The study started in 2011, the first year of JENCI online publishing by Elsevier. The study ended in 2016 being the last complete year of JENCI’s publications at the time approving the study protocol. All online-available original research articles published during this period were included. Other published research types during the same period; case reports, reviews, and letters to editors were not included in the current study.

### The data collection form

It consisted of two parts:

The first part included the general article information, e.g., serial number, article title, and specialty as well as the institution, and year of publication, while the second part included the reviewed items of every article’s section from title to references as follows:

#### Title

Clarity of reporting (adequate study description, clear and simple phrasing without unfamiliar terms (jargons), uncommon or un-declared abbreviations, with precise description and relevance to study), absence of abbreviations or formula, and reflecting the aim with clear mention of the study design.

#### Abstract

Clarity of reporting that makes it stand-alone, concision, using a structured writing format (aim, methodology, results, and conclusion), and without references or abbreviations.

#### Introduction

This included the aim, rationale, relevant previous studies to the studied topic, and did not include material belonging to other sections of the manuscript (e.g., methods or results).

#### Methods

Sampling method reporting and type, randomization reporting and method (considered applicable multi-armed clinical trials), matching technique reporting and method (considered applicable in case-control studies), blinding documentation and its method (if used), PICOT components reporting (population, intervention, comparator group if applicable, outcome, and time), inclusion and exclusion criteria documentation, study scope (classified to either preventive, therapeutic, diagnostic, prognostic, basic science (physics, chemistry, biology, and mathematics), or others), study design and type, study timing and type, absence of material belonging to other sections of the manuscript, data collection tool reporting with comment on validity and reliability aspects (for questionnaires), statistical methods (reporting, appropriateness, completeness, including the significance level confidence interval (if applicable or not used), and correctly defining any used statistical variables (e.g., survival terms, morbidity, or mortality rates)), sample size estimation.

#### Results

Clarity of reporting ( accurate, complete, relevant, clearly phrased with no contradicting within the text), exhaustiveness (with post hoc tests and multivariate analysis whenever applicable), relevance to aim, answering the research question, and had no material belonging to other sections of the manuscript, appropriate use of statistical terms (e.g., correlation, prevalence or incidence, trend), appropriate selection or application of descriptive parameters, or statistical tests with correct statistical calculations.

#### Tables

Accurate titles and labels (complete, correct, and without misused statistical terms), completely presenting the applied statistical material (e.g., survival tables), informative (complete and correct information), emphasizing the written text (informative but not merely repeating the text or figure or contradicting them), without irrelevant or redundant information, all tables correctly referred to in the text, organized, and consistently formulated within each table and in-between tables.

#### Figures

Accurate titles and labels (correct, complete, and without misused statistical terms), informative (correct information, complete with clear figure formatting), organized, consistently formulated (if multiple), without contradiction with the written text or tables, emphasizing the written text (informative but not merely repeating the text or contradicting it), without irrelevant or redundant information, and all figures are correctly referred to in the text.

#### Discussion

Clarity of reporting (accurate, complete, relevant, clearly phrased with no contradictions within the text), correct interpretation complete coverage of the results, no comparison with non-similar studies, no material belonging to other sections of the manuscript, full justification of findings in relation to literature and evidence, no contradiction within text or extrapolation beyond results range, not redundant, followed a logical flow, and addressing strengths and limitations.

#### Conclusion

Clarity of reporting (accurate, relevant, clear phrasing with no contradicting within the text), short, precise, linked to title and aim, reasonable and logical, and justified by the results without extrapolation.

#### Recommendations

Reporting and clarity of statement.

#### References

Mostly recent (more than half of them within maximum 10 years of publication year), present for all cited texts, and list of references completely mentioned in the text.

#### Others

Abbreviations are fully mentioned at first use, and the state of any conflict of interest is reported.

### Statistical methods

Data management and analysis were performed using Statistical Package for Social Sciences (SPSS) vs. 25. Each item in the checklist was summarized as a number and percentage, and for each item, a score of 1 was awarded for the “YES; favorable method” and a score of 0 for the “NO; un-favorable method.” A summary score for the whole article and for each major subsection was computed by adding all scores of each item. The summary scores were tested for normality using Kolmogrov-Smirnov test and Shapiro-Wilk test. The medians and ranges were computed for the scores as they were non-normally distributed.

The whole study period between 2011 and 2016 was divided into two periods early: from 2011 to 2013 and recent from 2014 to 2016. Each item in the checklist was compared using chi-square or Fisher’s exact test as appropriate. The summary scores of all reviewed sections from title to references were compared between the 2 time periods using the Student’s *t* test, in case they were normally distributed and by the Mann-Whitney test, if not normally distributed. All statistical tests were two-sided. The threshold of statistical significance of the *p* value was fixed at the 5% level.

## Results

### General article information

Throughout the studied period, clinical pathology specialty had the highest participation (41%). The majority of the articles (81.3%) were a single-centre work. Although the 6 years had more or less a comparable number of publications, the highest percent of publications occurred in 2014 (19.4%) while the lowest percent occurred in 2016 (14.4%), Table 1.

**Table 1:** Characteristics of the reviewed original articles (*n* = 139) published in the *Journal of the Egyptian National Cancer Institute*, in the period from 2011 to 2016

Item		Number (%)
**Specialty**	Clinical pathology	57 (41.0)
	Medical oncology	32 (23.0)
	Surgical oncology	28 (20.1)
	Radiation oncology	14 (10.1)
	Pediatric oncology	6 (4.3)
	Others (Epidemiology and Pharmacology)	2 (1.4)
**Centers**	Mentioned	123 (88.5)
	Single center	113 (91.9)
	Two centers	9 (7.3)
	More than two centres	1 (0.8)
	Not mentioned	16 (11.5)
**Publication year**	2011	23 (16.5)
	2012	23 (16.5)
	2013	25 (18.0)
	2014	27 (19.4)
	2015	21 (15.1)
	2016	20 (14.4)

### Description of all items per sections of the reviewed articles

#### Title, abstract, and introduction section

About 60% of titles were clearly stated, 83.5% of them had no abbreviations or formulae but only 51.1% of them represented the aim and 9.4% represented the study design. Abstract sections were concise, without references, and had structured writing format in the majority of articles (98.6%, 99.3%, and 93.5%, respectively), while only 35.3% of abstracts provided a clear study overview and 50.4% had no abbreviations or formulae. The introduction section included the study aim or objectives in 91.4%, relevant previous studies in 92.8%, no material belonging to other section(s) in 97.8%, while only 41% of them included the study rationale.

#### Methods’ section

Table 2 depicts the results regarding study design, study timing, and study scope. The sampling method was reported in 127 articles; all of them used a non-random method either in the form of the consecutive time period technique in 98.4% (125/127) or judgmental technique in 1.6% (2/127).

**Table 2:** Study design, timing, and scope of the reviewed original articles (*n* = 139) published in the *Journal of the Egyptian National Cancer Institute*, in the period from 2011 to 2016

Item		Number (%)
**Study design**		
Clearly stated		**36 (25.9)**
Type	Case-control	11 (30.6)
	Cohort	10 (27.7)
	Cross sectional	5 (13.9)
	Clinical trial	5 (13.9)
	Pilot	3 (8.3)
	Documentary	1 (2.8)
	Case series	1 (2.7)
**Study timing**		
Reported		**82 (59.0)**
	Retrospective	54 (65.8)
Timing	Prospective	19 (23.2)
	Non-directional	5 (6.1)
	Combined	4 (4.9)
**Study scope**	Prognostic	44 (31.7)
	Therapeutic	34 (24.5)
	Diagnostic	30 (21.6)
	Basic science	17 (12.2)
	Epidemiologic	9 (6.5)
	Preventive	4 (2.9)
	Others	1 (0.7)

Regarding PICOT reporting, all articles reported their populations, their interventions (if present), and all applicable articles reported the comparators. Only 88.5% of articles reported their outcomes and 89.9% of the applicable articles reported their study time. Inclusion criteria and exclusion criteria were clearly reported in 43.1% and 40.1% of the applicable studies, respectively.

Out of the 4 applicable articles for randomization, 18 applicable articles for matching and 4 applicable articles for blinding, 2 reported randomization (without mention of their method), 7 reported matching, and none reported blinding. Only 4 articles (2.9%) reported sample size estimation. The methods’ section included materials that belong to Results in 74.1% of the articles.

Questionnaires were only used in 2 articles but without any comment on their validity or reliability aspects. Statistical methods were described in 80.6% of the articles. Although 9.4% of articles reported statistical methods without using them, most reported methods were appropriate (85.7%), Table 3

**Table 3:** Statistical methodology of the reviewed original articles (*n* = 139) published in the *Journal of the Egyptian National Cancer Institute*, in the period from 2011 to 2016

Item	Number (%)
**Statistical methods**	
Described	**112 (80.6)**
Appropriate	96 (85.7)
Complete	34 (30.4)
Level of significance defined (*n* = 104) *	92 (88.5)
Confidence interval determined (*n* = 58)**	17 (29.3)
Statistical variables correctly defined (*n* = 51)***	23 (45.1)

*P value was not used or significance level was not applicable in 8 articles

**Confidence interval was not used or not applicable in 54 articles

***Statistical variables were not used or not applicable in 61 articles

#### Results’ section

About 37% of articles had clearly reported their results, 52.5% had them exhaustive, and 58.3% were answering the targeted research question. Thirty-nine articles had materials that did not belong to results. Misused statistical terms were found in 16.5%, incorrect statistical calculations in 41.0%, irrelevant results in 21.6%, and inappropriate statistical tests or descriptive parameters selection in 26.6%, and out of the 110 applicable studies, inappropriate application of tests was found in 54 articles. Reasons for inappropriate selection and application of tests are shown in Table 4 and Table 5, respectively.

**Table 4:** Reasons for inappropriate selection of statistical tests or descriptive parameters found in 37 articles published in the *Journal of the Egyptian National Cancer Institute*, in the period from 2011 to 2016

Error	Number (%)
**Misused descriptive parameters**	
Mean for categorical data	2 (5.4)
Mean for Non-normally distributed data or scores	20 (54.1)
Mean survival or mean follow up time	7 (18.9)
Percent surviving	2 (5.4)
Range or Interquartile range instead standard deviation	3 (8.1)
**Misused statistical analysis**	3 (8.1)
(McNemar, *T* test and logistic regression)

**Table 5:** Reasons for inappropriate application of statistical tests found in 54 articles published in the *Journal of the Egyptian National Cancer Institute*, in the period from 2011 to 2016

Error	Number (%)
**Single misapplied test**	**52 (96.3)**
Small expected number for Chi-square test	30 (55.6)
Univariate logistic or cox regression	4 (7.4)
Compare survival between respondent and non-respondents	5 (9.3)
Calculate diagnostic accuracy with no or inaccurate gold standard	3 (5.6)
Combine different groups in analysis	3 (5.6)
Include unknown as a category in analysis	2 (3.7)
*T* test, ANOVA, or correlation for categorical or non-parametric data	5 (9.3)
**Multiple misapplied tests**	2 (3.7)

*ANOVA*: analysis of variance

**Table 6:** Tabulated and graphically presented data of the reviewed original articles (*n* = 139) published in the *Journal of the Egyptian National Cancer Institute*, in the period from 2011 to 2016

Item	Number (%)
Tables	
Present	128 (92.1)
Accurate titles and labels	24 (18.8)
Completely presenting applied statistical material	86 (67.2)
Independently informative	68 (53.1)
Complete statistical data	62 (48.4)
Correct statistical data	64 (50.0)
Organized tables	48 (37.5)
Not repeating figures information (*n* = 115)*	112 (97.4)
Emphasizing written text	71 (55.5)
Not contradicting written text	96 (75.0)
Relevant and not redundant information	90 (70.3)
Consistent formulation within each table	61 (47.7)
Consistent formulation in-between tables (*n* = 116)**	51 (44.0)
All tables correctly referred to in the text	109 (85.2)
Figures	
Present	82 (59.0)
Accurate titles and labels	16 (19.5)
Independently informative	34 (41.5)
Organized	52 (63.4)
Emphasizing written text	34 (41.5)
Not contradicting written text	76 (92.7)
Relevant and not redundant	58 (70.7)
Consistent formulation in-between (*n* = 67)***	55 (67.1)
All figures correctly referred to in the text	74 (90.2)

*13 studies had no statistical figures and were considered inapplicable for this item

**12 studies had a single table and were considered inapplicable for this item

***15 studies had a single figure and were considered inapplicable for this item

Only 92.1% of articles included statistical tables (single table in 9.4% while multiple in 90.6%). Although the majority of tables had relevant and non-redundant data, they were correctly referred to in text. Only 59.0% of articles had statistical figures (single figure in 18.3% while multiple in 81.7%). Most figures did not contradict the written text, had relevant and non-redundant data, and were correctly referred to in text, Table 6.

#### Discussion section

Most discussion sections were unclearly reported (69.8%) with incomplete coverage the results (60.4%), and 53.5% of them correctly interpreted the results. About 85% of them had redundant or irrelevant information, 56.8% used un-similar studies for comparison, and 25.9% extrapolated beyond their results’ range. Only 31.7% of discussion sections fully justified the findings, and 56.1% of them followed a logical flow of reporting. The contradiction within the text was observed in 36.7%.

#### Conclusions section and others

Seven articles had no conclusion section. Most conclusions were short (93.9%), linked to aim and title (82.6%), and reasonable and logical (56.1%), but less likely to be precise (22.7%), clearly stated (45.5%) or limited to the results without extrapolation (29.5%). Seventy-eight articles (56.1 %) reported the study recommendations; most of them were clearly stated (80.8%). Citation lists were completely mentioned in the text in 91.4%, recent in 64.7%, and present for all cited text in 61.2%. Only 29.5% of abbreviations were fully mentioned at first use. Only 85 articles mentioned their state of conflict of interest (all of them reported the absence of any conflict).

To summarize, the main pitfalls in the present study were as follows: *Titles* were unclear in 40.0%, they did not reflect the study aim in 48.9% and did not include the study design in 90.6%. *Abstracts* did not provide a clear study overview in 64.7%, while 59.0% of *introduction* sections did not include a study rationale. In *methods’* sections, 56.9% and 59.9% of articles did not report inclusion and exclusion criteria, respectively, 97.1% did not report sample size estimation, 19.4% did not report their statistical methods at all, and in case of reporting them, 14.3% of them were inappropriate and 69.6% were incomplete. The confidence interval was undetermined in 70.7%, and statistical variables were incorrectly defined in 54.9%. Regarding *results’* sections, 63.0% of them were unclearly reported, 47.5% were non-exhaustive, 41.7% did not answer research question, 41.0% did incorrect statistical calculations, and 49.1% inappropriately applies their tests. *Tables* had inaccurate titles or labels in 81.2%, incomplete statistical data in 51.6%, incorrect data in 50.0%, and were unorganized in 62.5%. *Figures* had inaccurate titles or labels in 80.5%, not emphasizing written text in 58.5%, and were independently non-informative in 58.5%. In *discussion* sections, 69.8% were unclearly reported, 60.4% incompletely covered results, 46.5% incorrectly interpreted results, 56.8% compared with un-similar studies, 25.9% extrapolated beyond results, 68.3% incompletely justified the findings, and contradiction within the text was observed in 36.7%. Most articles also lack reporting their study limitations and strengths. Only 22.7%, 45.5%, and 29.5% of *conclusions’* sections were precise, clearly stated, and did not extrapolate beyond results, respectively.

### Comparison between the early and recent study periods regarding all items and sections of the reviewed articles

Statistical comparison between the early (*n* = 71) and recent (*n* = 68) periods regarding all reviewed items showed a significant decrease in misusing statistical terms (23.9% in early versus 8.8% in the recent period, *p* value = 0.016). A significant increase was found in reporting the state of conflict of interest (95.6% in recent versus 28.2% in the early period, *p* value < 0.001) and a significant decrease in the percent of complete references for all cited texts (51.5% in recent versus 70.4% in the early period, *p* value = 0.022). Besides, a statistically significant increase in NCI participation (56.9%) over the other institutes (43.1%) in the early period, while the opposite was observed in the recent period; 36.2% versus 63.8%, respectively (*p* value = 0.022). Nothing else was significantly different between both periods. No statistically significant difference was found between both periods regarding the calculated median scores.

## Discussion

The ultimate goal of this study was to review previous patterns in research published at JENCI, from 2011 to 2016 to be used to maximize the achievements and minimize the shortcomings. Efforts of all authors were appreciated; however, errors in analyzing and reporting research were very common.

Despite its vital importance, the study design was clearly mentioned in only 25.9% of articles in the current study, compared to 25.7% by Allam et al. study [4], and this stressed the need to involve a biostatistician at the very early stages of the study.

Statistical methods were not described 19.4%, in incomplete in 69.6%, and inappropriate in 14.3 of the articles. Using incorrect or incomplete statistical methods can produce misleading, suboptimal, or incoherent results available to be cited by other researchers [14]. Allam et al. [4] also reported absent, inappropriate, and incomplete statistical methods in 14.5%, 75.5%, and 47.2%, respectively. Common statistical misuse might be explained by lacking basic statistical knowledge among the medical community in general [15]. On the contrary, 9.4% reported statistical methods without using them in this study. Ercan et al. [14] reported a comparable percent (6.08%), while Hanif and Ajmal [16] reported a higher percent (21.3%). Consistent and objective rules guiding authors to report their research should be strictly applied.

Only 4 articles of the current study and 4 dissertations in Allam et al. [4] study had a basis of sample size estimation. Inclusion criteria and exclusion criteria were clearly reported in 43.1% and 40.1% of the applicable 137 articles in the current study corresponding to 79.0% combined for both in Allam et al. [4] study.

In the current study, 47.5% had non-exhaustive results, 16.5% had misused statistical terms, 26.6% used inappropriate statistical tests, and 41.0% had incorrect statistical calculations. Ercan et al. [14] reported a lower rate of non-exhaustive results (26.5%), a similar rate regarding misused statistical terms (20.4%), incorrect statistical calculations (43.6%), and inappropriate statistical tests (28.2%). Hanif and Ajmal [16] also reported a comparable rate of inappropriate statistical tests (28.8%), Allam et al. [4] and Šimundić and Nikolac [17] reported higher rates (53.2% and 61.8%, respectively), while Karan et al. [18] reported a lower rate (7.0%).

In the present study, the overall rate of inappropriate interpretation of statistical analysis results was 46.5% compared to 10.5%, 13.8%, 18%, and 82.3% in Ercan et al. [14], Hanif and Ajmal [16], Bakker and Wicherts [19], and Allam et al. [4] studies, respectively. Misinterpreting the results may ruin the deduced conclusions, thus drawing conclusions should be sufficiently supported by the data should be avoided, Strasak et al. [20].

On comparing the recent and early periods, a statistically significant less misuse of statistical terms, more reporting the state of conflict of interest, and less complete references for all cited texts were found between both periods, respectively. A significant increase in NCI participation over other institutes in the early period while the opposite occurred in the recent period was also noticed. Nothing else was significantly different between both periods. The inability to show a significant improvement overtime in most items could be explained by the significant less participation of NCI over other institutions in the recent period and more participation in the early period, because NCI, unlike other institutions, had a specialized team of committed epidemiologists to provide the methodological and statistical guidance, revise protocols, and estimate sample sizes for any research all the time.

### Study strengths and limitations

Being the first study reviewing articles in JENCI or other Egyptian journals is a major point of strength. The online accessibility, the diversity of literature published in JENCI, the development of the collective checklist applicable to most study types, and the wide experience of the study supervisors in the field of critical appraisal had also added to the value of this study.

Study limitations were being a non-comprehensive study that did not show the overall picture of research at JENCI (as the certain time period from 2011 to 2016 was specified and years prior to or after that period were not involved), the small number of published multi-armed randomized clinical trials that hindered accurate assessment of randomization and blinding. The scarcity of similar studies in the Egyptian literature to compare with in the discussion section was also challenging.

## Conclusion

The articles published in JENCI (from 2011 to 2016) had many methodological and reporting defects that may compromise the power of the results and their external validity. To overcome these consequences, continuous training of researchers on the basics of epidemiology, biostatistics, and research methodology is highly recommended. This training could be done through adding courses for the undergraduate student, applying research methodology in small projects for graduation, or refreshing lectures, and workshops for postgraduate students and staff members. Involving well-trained epidemiologists from the early beginning and throughout the whole research and publication process in JENCI would ensure a good preparation, implementation, and reporting of research. Following the scientific reporting guidelines as CONSORT guidelines for clinical trials or STROBE guidelines for observational studies can ensure a complete, organized, and of high-quality scientific material. On comparing early (2011–2013) and recent (2014–2016) periods, only a significant less misuse of statistical terms, more reporting conflict of interest, more missing references for cited texts in the recent period, and more participation of NCI over other institutes in the early period were found.

## Data Availability

All reviewed articles are of open access and available online.

## Abbreviations

JENCI: *Journal of the Egyptian National Cancer Institute*
PICOT: Population, Intervention, Comparison, Outcomes, and Time
ANOVA: Analysis of variance
